# *N*-(3-Nitro­benzo­yl)benzene­sulfonamide

**DOI:** 10.1107/S1600536812016765

**Published:** 2012-04-21

**Authors:** P. A. Suchetan, Sabine Foro, B. Thimme Gowda

**Affiliations:** a Department of Chemistry, Mangalore University, Mangalagangotri 574199, Mangalore, India; b Institute of Materials Science, Darmstadt University of Technology, Petersenstrasse 23, D-64287, Darmstadt, Germany

## Abstract

In the title compound, C_13_H_10_N_2_O_5_S, the C=O bond in the —SO_2_—NH—CO— segment is *anti* to the *meta*-nitro group in the benzoyl ring, while the N—C bond has *gauche* torsions with respect to the S=O bonds. The molecule is twisted at the N atom with a dihedral angle of 79.9 (2)° between the sulfonyl benzene ring and the —SO_2_—NH—CO— segment. Furthermore, the dihedral angle between the benzeneline rings is 86.9 (2)°. In the structure, the mol­ecules are linked into helical chains along the *b* axis *via* N—H⋯O hydrogen bonds.

## Related literature

For our studies on the effects of substituents on the structures and other aspects of *N*-(ar­yl)-amides, see: Gowda *et al.* (2000 ▶, 2007 ▶), of *N*-(substitutedbenzo­yl)-aryl­sulfonamides, see: Gowda *et al.* (2009 ▶), of *N*-chloro­aryl­amides, see: Jyothi & Gowda (2004 ▶) and of *N*-bromo­aryl­sulfonamides, see: Usha & Gowda (2006 ▶).
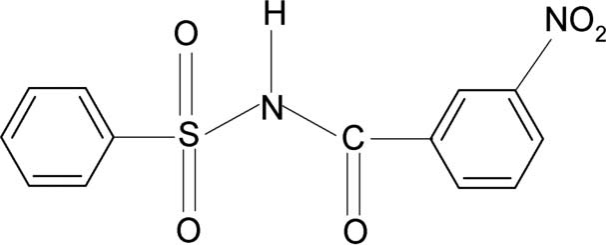


## Experimental

### 

#### Crystal data

C_13_H_10_N_2_O_5_S
*M* *_r_* = 306.29
Orthorhombic, 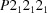

*a* = 5.1053 (5) Å
*b* = 13.078 (1) Å
*c* = 20.163 (2) Å
*V* = 1346.2 (2) Å^3^
*Z* = 4
Mo *K*α radiation
μ = 0.26 mm^−1^
*T* = 293 K
0.48 × 0.12 × 0.12 mm

#### Data collection

Oxford Diffraction Xcalibur diffractometer with Sapphire CCD Detector
Absorption correction: multi-scan *CrysAlis RED* (Oxford Diffraction, 2009 ▶) *T*_min_ = 0.884, *T*_max_ = 0.969
4590 measured reflections
2282 independent reflections
1869 reflections with *I* > 2σ(*I*)
*R*_int_ = 0.028

#### Refinement

*R*[*F* ^2^ > 2σ(*F* ^2^)] = 0.065
*wR*(*F* ^2^) = 0.137
*S* = 1.38
2282 reflections
193 parameters
1 restraint
H atoms treated by a mixture of independent and constrained refinement
Δρ_max_ = 0.28 e Å^−3^
Δρ_min_ = −0.32 e Å^−3^
Absolute structure: Flack (1983 ▶), 871 Friedel pairs
Flack parameter: −0.1 (2)

### 

Data collection: *CrysAlis CCD* (Oxford Diffraction, 2009 ▶); cell refinement: *CrysAlis RED* (Oxford Diffraction, 2009 ▶); data reduction: *CrysAlis RED*; program(s) used to solve structure: *SHELXS97* (Sheldrick, 2008 ▶); program(s) used to refine structure: *SHELXL97* (Sheldrick, 2008 ▶); molecular graphics: *PLATON* (Spek, 2009 ▶); software used to prepare material for publication: *SHELXL97*.

## Supplementary Material

Crystal structure: contains datablock(s) I, global. DOI: 10.1107/S1600536812016765/bt5879sup1.cif

Structure factors: contains datablock(s) I. DOI: 10.1107/S1600536812016765/bt5879Isup2.hkl

Supplementary material file. DOI: 10.1107/S1600536812016765/bt5879Isup3.cml

Additional supplementary materials:  crystallographic information; 3D view; checkCIF report

## Figures and Tables

**Table 1:** Hydrogen-bond geometry (Å, °)

*D*—H⋯*A*	*D*—H	H⋯*A*	*D*⋯*A*	*D*—H⋯*A*
N1—H1*N*⋯O2^i^	0.86 (2)	2.05 (2)	2.909 (6)	175 (6)

Symmetry code: (i) 
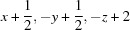
.

**Fig. 1.**
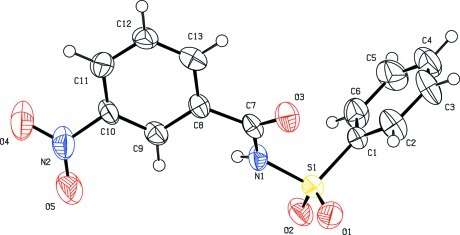
Molecular structure of the title compound, showing the atom- labelling scheme. Displacement ellipsoids are drawn at the 50% probability level.

**Fig. 2.**
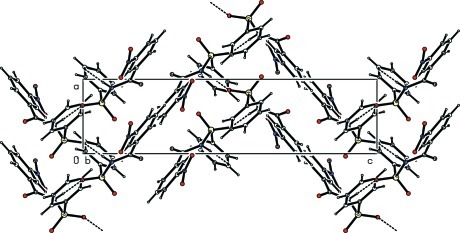
Molecular packing in the title compound. Hydrogen bonds are shown as dashed lines.

## supplementary crystallographic information

### Comment

Diaryl acylsulfonamides are known as potent antitumor agents against a broad spectrum of human tumor xenografts in nude mice. As part of our studies on the substituent effects on the structures and other aspects of *N*-(aryl)-amides (Gowda *et al.*, 2000, 2007), *N*-(substitutedbenzoyl)-arylsulfonamides (Gowda *et al.*, 2009), *N*-chloroarylsulfonamides (Jyothi & Gowda, 2004) and *N*-bromoarylsulfonamides (Usha & Gowda, 2006), in the present work, the crystal structure of *N*-(3-nitrobenzoyl)benzenesulfonamide has been determined (Fig.1).

The conformation of the N—H bond in the C—SO_2_—NH—C(O) segment is *anti* to the C=O bond (Fig.1), similar to that observed in *N*-(3-chlorobenzoyl)benzenesulfonamide (I)(Gowda *et al.*, 2009).

Further, the C=O bond in the segment is *anti* to the *meta*-nitro group in the benzoyl ring, while the conformation of the N—C bond in the C—SO_2_—NH—C(O) segment of the structure has "*gauche*" torsions with respect to the S═O bonds. The molecule is twisted at the N atom with a dihedral angle of 79.9 (2)° between the sulfonyl benzene ring and the C—SO_2_—NH—C—O segment, compared to the value of 79.6 (1)° in (I).

The dihedral angles between the sulfonyl and the benzoyl benzene rings is 86.9 (2)°, compared to the value of 89.3 (1)° in (I).

The packing of molecules linked by of N—H···O(S) hydrogen bonds (Table 1) is shown in Fig. 2.

### Experimental

The title compound was prepared by refluxing a mixture of 3-nitrobenzoic acid, benzene sulfonamide and phosphorous oxy chloride for 5 h on a water bath. The resultant mixture was cooled and poured into ice cold water. The solid obtained was filtered, washed thoroughly with water and then dissolved in sodium bicarbonate solution. The compound was later reprecipitated by acidifying the filtered solution with dilute HCl. The filtered and dried solid was recrystallized to the constant melting point.

Rod like colourless single crystals of the title compound used in X-ray diffraction studies were obtained from a slow evaporation of the solvent from its toluene solution at room temperature.

### Refinement

The H atom of the NH group was located in a difference map and later restrained to N—H = 0.86 (2) %A. The other H atoms were positioned with idealized geometry using a riding model with C—H = 0.93 Å. All H atoms were refined with isotropic displacement parameters set to 1.2 times of the *U*_eq_ of the parent atom.

### Crystal data

**Table:**

C_13_H_10_N_2_O_5_S	*F*(000) = 632
*M**_r_* = 306.29	*D*_x_ = 1.511 Mg m^−^^3^
Orthorhombic, *P*2_1_2_1_2_1_	Mo *K*α radiation, λ = 0.71073 Å
Hall symbol: P 2ac 2ab	Cell parameters from 1855 reflections
*a* = 5.1053 (5) Å	θ = 3.0–27.8°
*b* = 13.078 (1) Å	µ = 0.26 mm^−^^1^
*c* = 20.163 (2) Å	*T* = 293 K
*V* = 1346.2 (2) Å^3^	Rod, colourless
*Z* = 4	0.48 × 0.12 × 0.12 mm

### Data collection

**Table:**

Oxford Diffraction Xcalibur diffractometer with Sapphire CCD Detector	2282 independent reflections
Radiation source: fine-focus sealed tube	1869 reflections with *I* > 2σ(*I*)
Graphite monochromator	*R*_int_ = 0.028
Rotation method data acquisition using ω and phi scans.	θ_max_ = 25.0°, θ_min_ = 3.1°
Absorption correction: multi-scan *CrysAlis RED* (Oxford Diffraction, 2009)	*h* = −3→6
*T*_min_ = 0.884, *T*_max_ = 0.969	*k* = −15→11
4590 measured reflections	*l* = −20→24

### Refinement

**Table:**

Refinement on *F*^2^	Secondary atom site location: difference Fourier map
Least-squares matrix: full	Hydrogen site location: inferred from neighbouring sites
*R*[*F*^2^ > 2σ(*F*^2^)] = 0.065	H atoms treated by a mixture of independent and constrained refinement
*wR*(*F*^2^) = 0.137	*w* = 1/[σ^2^(*F*_o_^2^) + (0.*P*)^2^ + 2.6443*P*] where *P* = (*F*_o_^2^ + 2*F*_c_^2^)/3
*S* = 1.38	(Δ/σ)_max_ < 0.001
2282 reflections	Δρ_max_ = 0.28 e Å^−^^3^
193 parameters	Δρ_min_ = −0.32 e Å^−^^3^
1 restraint	Absolute structure: Flack (1983), 871 Friedel pairs
Primary atom site location: structure-invariant direct methods	Flack parameter: −0.1 (2)

### Special details

**Table:**

Experimental. Empirical absorption correction using spherical harmonics, implemented in SCALE3 ABSPACK scaling algorithm.
Geometry. All e.s.d.'s (except the e.s.d. in the dihedral angle between two l.s. planes) are estimated using the full covariance matrix. The cell e.s.d.'s are taken into account individually in the estimation of e.s.d.'s in distances, angles and torsion angles; correlations between e.s.d.'s in cell parameters are only used when they are defined by crystal symmetry. An approximate (isotropic) treatment of cell e.s.d.'s is used for estimating e.s.d.'s involving l.s. planes.
Refinement. Refinement of *F*^2^ against ALL reflections. The weighted *R*-factor *wR* and goodness of fit *S* are based on *F*^2^, conventional *R*-factors *R* are based on *F*, with *F* set to zero for negative *F*^2^. The threshold expression of *F*^2^ > σ(*F*^2^) is used only for calculating *R*-factors(gt) *etc*. and is not relevant to the choice of reflections for refinement. *R*-factors based on *F*^2^ are statistically about twice as large as those based on *F*, and *R*- factors based on ALL data will be even larger.

### Fractional atomic coordinates and isotropic or equivalent isotropic displacement parameters (Å^2^)

**Table:**

	*x*	*y*	*z*	*U*_iso_*/*U*_eq_	
C1	0.3551 (11)	0.4936 (4)	0.9411 (3)	0.0341 (12)	
C2	0.3105 (16)	0.5680 (5)	0.8942 (3)	0.0559 (18)	
H2	0.1925	0.5564	0.8599	0.067*	
C3	0.4420 (17)	0.6597 (5)	0.8985 (4)	0.067 (2)	
H3	0.4091	0.7108	0.8675	0.080*	
C4	0.6203 (16)	0.6760 (5)	0.9478 (4)	0.064 (2)	
H4	0.7113	0.7375	0.9498	0.077*	
C5	0.6647 (16)	0.6020 (5)	0.9941 (4)	0.067 (2)	
H5	0.7854	0.6137	1.0278	0.081*	
C6	0.5320 (14)	0.5097 (5)	0.9915 (3)	0.0539 (18)	
H6	0.5620	0.4595	1.0233	0.065*	
C7	0.4855 (12)	0.3009 (4)	0.8392 (3)	0.0362 (14)	
C8	0.6560 (12)	0.2129 (4)	0.8190 (3)	0.0351 (13)	
C9	0.6177 (11)	0.1146 (4)	0.8425 (3)	0.0389 (14)	
H9	0.4848	0.1004	0.8727	0.047*	
C10	0.7814 (13)	0.0385 (4)	0.8202 (3)	0.0398 (15)	
C11	0.9833 (13)	0.0555 (5)	0.7769 (3)	0.0456 (16)	
H11	1.0941	0.0026	0.7642	0.055*	
C12	1.0182 (13)	0.1533 (4)	0.7527 (3)	0.0450 (16)	
H12	1.1517	0.1669	0.7226	0.054*	
C13	0.8550 (13)	0.2305 (5)	0.7732 (3)	0.0450 (16)	
H13	0.8779	0.2960	0.7562	0.054*	
N1	0.3859 (10)	0.2927 (3)	0.9027 (2)	0.0383 (12)	
H1N	0.458 (11)	0.256 (4)	0.933 (2)	0.046*	
N2	0.7308 (14)	−0.0663 (4)	0.8455 (3)	0.0612 (18)	
O1	−0.0348 (8)	0.3898 (3)	0.8930 (2)	0.0584 (13)	
O2	0.1461 (10)	0.3402 (3)	1.0022 (2)	0.0561 (13)	
O3	0.4341 (9)	0.3708 (3)	0.8029 (2)	0.0504 (11)	
O4	0.9051 (11)	−0.1293 (4)	0.8380 (3)	0.0777 (16)	
O5	0.5233 (14)	−0.0835 (4)	0.8715 (4)	0.109 (3)	
S1	0.1833 (3)	0.37784 (11)	0.93608 (8)	0.0406 (4)	

### Atomic displacement parameters (Å^2^)

**Table:**

	*U*^11^	*U*^22^	*U*^33^	*U*^12^	*U*^13^	*U*^23^
C1	0.036 (3)	0.026 (3)	0.040 (3)	−0.003 (2)	0.001 (3)	−0.003 (3)
C2	0.077 (5)	0.043 (4)	0.048 (4)	−0.011 (4)	−0.011 (4)	0.002 (3)
C3	0.101 (6)	0.031 (4)	0.069 (5)	−0.013 (4)	−0.007 (5)	0.012 (3)
C4	0.076 (5)	0.040 (4)	0.077 (5)	−0.016 (4)	0.000 (5)	−0.007 (4)
C5	0.064 (5)	0.060 (5)	0.077 (5)	−0.019 (4)	−0.024 (4)	−0.011 (4)
C6	0.057 (4)	0.044 (4)	0.060 (4)	0.003 (4)	−0.020 (4)	0.005 (3)
C7	0.045 (4)	0.025 (3)	0.039 (3)	−0.007 (3)	−0.008 (3)	0.001 (3)
C8	0.043 (3)	0.029 (3)	0.033 (3)	−0.004 (3)	−0.003 (3)	−0.004 (2)
C9	0.047 (4)	0.035 (3)	0.034 (3)	−0.007 (3)	0.007 (3)	−0.003 (3)
C10	0.052 (4)	0.023 (3)	0.044 (3)	−0.006 (3)	0.005 (3)	0.000 (3)
C11	0.047 (4)	0.042 (4)	0.048 (4)	0.001 (3)	0.010 (3)	−0.013 (3)
C12	0.046 (4)	0.043 (4)	0.046 (3)	−0.008 (3)	0.018 (3)	−0.003 (3)
C13	0.049 (4)	0.046 (4)	0.041 (3)	−0.016 (3)	−0.001 (3)	0.001 (3)
N1	0.047 (3)	0.027 (2)	0.041 (3)	0.007 (2)	0.000 (2)	0.005 (2)
N2	0.089 (6)	0.038 (3)	0.057 (4)	0.012 (3)	0.019 (4)	−0.002 (3)
O1	0.040 (2)	0.048 (3)	0.087 (3)	0.000 (2)	−0.012 (2)	−0.016 (3)
O2	0.074 (3)	0.038 (2)	0.056 (3)	−0.007 (2)	0.022 (3)	0.002 (2)
O3	0.065 (3)	0.040 (2)	0.046 (2)	0.005 (2)	−0.005 (2)	0.012 (2)
O4	0.111 (4)	0.047 (3)	0.074 (3)	0.029 (4)	0.020 (3)	0.007 (3)
O5	0.123 (6)	0.045 (3)	0.160 (6)	0.004 (3)	0.088 (5)	0.019 (3)
S1	0.0403 (8)	0.0314 (7)	0.0502 (8)	−0.0042 (7)	0.0052 (8)	−0.0019 (7)

### Geometric parameters (Å, º)

**Table:**

C1—C2	1.376 (8)	C8—C13	1.391 (8)
C1—C6	1.376 (8)	C9—C10	1.376 (8)
C1—S1	1.753 (5)	C9—H9	0.9300
C2—C3	1.377 (9)	C10—C11	1.368 (8)
C2—H2	0.9300	C10—N2	1.485 (8)
C3—C4	1.365 (10)	C11—C12	1.381 (8)
C3—H3	0.9300	C11—H11	0.9300
C4—C5	1.363 (9)	C12—C13	1.372 (8)
C4—H4	0.9300	C12—H12	0.9300
C5—C6	1.386 (9)	C13—H13	0.9300
C5—H5	0.9300	N1—S1	1.662 (5)
C6—H6	0.9300	N1—H1N	0.86 (2)
C7—O3	1.200 (7)	N2—O5	1.203 (8)
C7—N1	1.382 (7)	N2—O4	1.222 (7)
C7—C8	1.499 (8)	O1—S1	1.421 (4)
C8—C9	1.385 (8)	O2—S1	1.433 (4)
			
C2—C1—C6	120.6 (6)	C8—C9—H9	120.9
C2—C1—S1	119.2 (5)	C11—C10—C9	123.3 (5)
C6—C1—S1	120.2 (5)	C11—C10—N2	120.0 (6)
C1—C2—C3	119.5 (6)	C9—C10—N2	116.7 (5)
C1—C2—H2	120.3	C10—C11—C12	118.2 (6)
C3—C2—H2	120.3	C10—C11—H11	120.9
C4—C3—C2	120.4 (7)	C12—C11—H11	120.9
C4—C3—H3	119.8	C13—C12—C11	119.7 (6)
C2—C3—H3	119.8	C13—C12—H12	120.1
C5—C4—C3	120.0 (7)	C11—C12—H12	120.1
C5—C4—H4	120.0	C12—C13—C8	121.5 (6)
C3—C4—H4	120.0	C12—C13—H13	119.3
C4—C5—C6	120.7 (7)	C8—C13—H13	119.3
C4—C5—H5	119.6	C7—N1—S1	123.5 (4)
C6—C5—H5	119.6	C7—N1—H1N	123 (4)
C1—C6—C5	118.8 (6)	S1—N1—H1N	111 (4)
C1—C6—H6	120.6	O5—N2—O4	124.7 (6)
C5—C6—H6	120.6	O5—N2—C10	118.4 (6)
O3—C7—N1	122.9 (6)	O4—N2—C10	116.9 (6)
O3—C7—C8	123.1 (5)	O1—S1—O2	120.2 (3)
N1—C7—C8	114.0 (5)	O1—S1—N1	108.3 (3)
C9—C8—C13	118.9 (6)	O2—S1—N1	103.2 (3)
C9—C8—C7	122.5 (5)	O1—S1—C1	109.4 (3)
C13—C8—C7	118.6 (5)	O2—S1—C1	108.0 (3)
C10—C9—C8	118.3 (5)	N1—S1—C1	106.9 (3)
C10—C9—H9	120.9		
			
C6—C1—C2—C3	−0.9 (10)	C11—C12—C13—C8	1.0 (10)
S1—C1—C2—C3	179.0 (6)	C9—C8—C13—C12	−2.0 (9)
C1—C2—C3—C4	1.7 (12)	C7—C8—C13—C12	179.9 (5)
C2—C3—C4—C5	−1.5 (12)	O3—C7—N1—S1	0.4 (8)
C3—C4—C5—C6	0.5 (13)	C8—C7—N1—S1	−177.7 (4)
C2—C1—C6—C5	−0.1 (10)	C11—C10—N2—O5	−164.3 (7)
S1—C1—C6—C5	180.0 (6)	C9—C10—N2—O5	15.9 (10)
C4—C5—C6—C1	0.3 (12)	C11—C10—N2—O4	15.4 (9)
O3—C7—C8—C9	−147.0 (6)	C9—C10—N2—O4	−164.4 (6)
N1—C7—C8—C9	31.1 (8)	C7—N1—S1—O1	54.4 (5)
O3—C7—C8—C13	31.0 (9)	C7—N1—S1—O2	−177.2 (5)
N1—C7—C8—C13	−150.9 (5)	C7—N1—S1—C1	−63.4 (5)
C13—C8—C9—C10	0.9 (8)	C2—C1—S1—O1	−16.3 (6)
C7—C8—C9—C10	178.8 (5)	C6—C1—S1—O1	163.6 (5)
C8—C9—C10—C11	1.4 (9)	C2—C1—S1—O2	−148.7 (5)
C8—C9—C10—N2	−178.8 (5)	C6—C1—S1—O2	31.2 (6)
C9—C10—C11—C12	−2.4 (9)	C2—C1—S1—N1	100.8 (5)
N2—C10—C11—C12	177.8 (6)	C6—C1—S1—N1	−79.3 (5)
C10—C11—C12—C13	1.2 (9)		

### Hydrogen-bond geometry (Å, º)

**Table:**

*D*—H···*A*	*D*—H	H···*A*	*D*···*A*	*D*—H···*A*
N1—H1*N*···O2^i^	0.86 (2)	2.05 (2)	2.909 (6)	175 (6)

Symmetry code: (i) *x*+1/2, −*y*+1/2, −*z*+2.
